# Early Life Events Carry Over to Influence Pre-Migratory Condition in a Free-Living Songbird

**DOI:** 10.1371/journal.pone.0028838

**Published:** 2011-12-16

**Authors:** Greg W. Mitchell, Christopher G. Guglielmo, Nathaniel T. Wheelwright, Corey R. Freeman-Gallant, D. Ryan Norris

**Affiliations:** 1 Department of Integrative Biology, University of Guelph, Guelph, Ontario, Canada; 2 Department of Biology, Advanced Facility for Avian Research, University of Western Ontario, London, Ontario, Canada; 3 Department of Biology, Bowdoin College, Brunswick, Maine, United States of America; 4 Department of Biology, Skidmore College, Saratoga Springs, New York, United States of America; University of Lethbridge, Canada

## Abstract

Conditions experienced during development can have long-term consequences for individual success. In migratory songbirds, the proximate mechanisms linking early life events and survival are not well understood because tracking individuals across stages of the annual cycle can be extremely challenging. In this paper, we first use a 13 year dataset to demonstrate a positive relationship between 1^st^ year survival and nestling mass in migratory Savannah sparrows (*Passerculus sandwichensis*). We also use a brood manipulation experiment to show that nestlings from smaller broods have higher mass in the nest relative to individuals from larger broods. Having established these relationships, we then use three years of field data involving multiple captures of individuals throughout the pre-migratory period and a multi-level path model to examine the hypothesis that conditions during development limit survival during migration by affecting an individual's ability to accumulate sufficient lean tissue and fat mass prior to migration. We found a positive relationship between fat mass during the pre-migratory period (Sept–Oct) and nestling mass and a negative indirect relationship between pre-migratory fat mass and fledging date. Our results provide the first evidence that conditions during development limit survival during migration through their effect on fat stores. These results are particularly important given recent evidence showing that body condition of songbirds at fledging is affected by climate change and anthropogenic changes to landscape structure.

## Introduction

Conditions experienced during early development, such as poor nutrition, can have long-term consequences for an individual's phenotype, subsequent performance, and fitness [1]–[3]. Many of these long-term effects occur over varying time scales and under specific ecological contexts [3]. In migratory animals, the annual cycle is comprised of a series of life history stages that often take place over broad geographical areas, allowing individuals to take advantage of seasonal fluctuations in resources [4], [5]. Critical to understanding how conditions during development affect future performance and fitness in migratory animals is being able to track individuals from development through successive life history stages [6], [7].

In many species of migratory songbirds, both experimental and natural variation in brood size can affect the condition of young in the nest, likely owing to either a decrease in available food per individual as brood size increases (6 of 9 species reviewed by [8], see also [9]–[13]) or to an increase in energy expenditure associated with begging as brood size increases [14], [15]. Similarly, the condition of young is also affected by natural and experimental variation in the timing of nesting, owing to either decreases in resource availability or parental provisioning rates as the breeding season progresses [13], [16], [17]. Furthermore, survival until the following breeding season (hereafter 1^st^ year survival) tends to be positively correlated with nestling mass (12 of 14 species reviewed; [8], [18], [19], see also [20]). Together, these studies suggest that conditions during development can affect 1^st^ year survival in migrants. However, because juveniles move large distances prior to migration [21], [22], are difficult to track during migration, and because natal dispersal can be broad in extent [23], [24], documenting the proximate mechanisms linking conditions during development with 1^st^ year survival has been challenging.

One mechanism that may lead to a positive correlation between the condition of young and 1^st^ year survival in migratory birds is if body condition in the nest carries forward into the pre-migratory period, when individuals need to build sufficient reserves to make long distance flights [25]–[27]. If young in poor condition are unable to compensate for their lower condition, then survival during migration may be compromised. In this paper, we first use a long-term dataset and a field experiment to show that nestling mass in a migratory population of Savannah sparrows is affected by nutritional or energetic constraints experienced during development and that 1^st^ year survival is strongly related to nestling mass. We then examine the hypothesis that conditions during development affect 1^st^ year survival through their effect on migratory body condition. Following this hypothesis, we predicted a positive relationship between pre-migratory lean tissue mass and pre-migratory fat mass with nestling mass. Using comprehensive experimental and observational data spanning multiple years and life-history stages, we provide the first evidence that conditions during development limit survival during migration through their effects on pre-migratory fat stores.

## Methods

### Study site and species

Fieldwork was conducted on Kent Island, an isolated 80 ha island in the Bay of Fundy, New Brunswick, Canada (44°35′N, 66°45′W). The northern third of the island is comprised of spruce-fir forest (*Picea glauca*, *P.rubens*, and *Abies balsamea*) and the southern two-thirds are characterized by old-field habitat. We studied a population of migratory Savannah sparrows inhabiting a 10 ha old-field located in the centre of the island. Savannah sparrows are grassland songbirds that breed in the northern United States and across Canada, and which mainly winter through the southern United States [28]. On Kent Island, median clutch size is four eggs (mean = 4.17, SD = 0.62, n = 1667 clutches; NTW unpublished data, [29]). Approximately 30% of breeding females on the island are double-brooded [30]. Nestlings typically fledge the nest at 9 d of age (range 9–11; [28], [31]). Both adults and juveniles begin departing the island from mid-September through late October (GWM, DRN, personal observation). Kent Island represents an ideal study site to examine early developmental conditions in a migratory species because its small size restricts the scope of pre-migratory movements, which facilitates the recapture of individuals prior to migration. Natal philopatry is also high (median dispersal distance = 228 m and <2 individuals per year were observed to have dispersed to surrounding islands across 10 years of census; [30]), allowing for a robust assessment of 1^st^ year apparent survival.

### Breeding history and capture

From 1989–1999, 2001, and 2008–2010, nests were located between 30 May–25 July by observing adult females during the nest building and incubation stages. When nestlings were 8 d old we measured mass using a top loading electronic balance (±0.1 g) and measured wing and tarsus lengths (±1 mm) according to Pyle [32]. From 1987–2001, 188 of 635 nests were found after hatch and from 2008–2010, three of 60 nests were found following hatch. For these nests, age was estimated using plumage characteristics and behavior [28]. Removal of these nests did not change the sign, magnitude, or overall significance of our final results, and so they were retained in our final analyses (Table S1, Table S2). Nests were not visited after nestlings were 8 d old to reduce the risk of premature fledging. We used the date of the last nest visit as our measure of timing of nesting.

In 2009 and 2010, we carried out experimental brood manipulations to establish a causal link between nestling condition and number of fledglings. In 2009, nests were both reduced (−1 nestling) and enlarged (+1 nestling). Only nests that were initially comprised of four nestlings were manipulated. Nests involved in the manipulation were paired based on hatching date, and one nest in the pair was randomly chosen for reduction. The nestling from the reduced nest (three nestlings) was transferred to the other nest, making it enlarged (five nestlings). Nestlings were 1 d of age at the time of transfer. The control group in our experiment consisted of all un-manipulated nests comprised of four nestlings. Analysis of the 2009 data suggested that the enlarged treatment did not have a significant effect on nestling mass relative to control broods (Table S3). Based on these results, we carried out only brood reductions in 2010. This was accomplished by removing one randomly chosen egg from a random subset of nests (Table S4).

To determine if early life events carry forward into the pre-migratory period, individuals were captured from 10 August–16 October in 2008, 31 August–19 October in 2009, and 18 August–21 October in 2010. Based on extensive field observations of foraging and social interactions and previous experimental work, all juveniles were independent of their family groups during this period (independence occurs by about 25 d of age; [33]). Juveniles were captured by flushing individuals from within a randomly chosen 50 m×50 m quadrat (29 in total) bordered on three sides by mist nets. All netting took place between 0700 and 1700 hr Atlantic Standard Time. Following capture, the same morphological measurements described for adults were taken for juveniles, as well as the condition measurements described below. Moult progression was assessed via the completeness of body moult and was scored as an ordinal variable (1≤25%, 2 = 25–49%, 3 = 50–74%, 4≥75%). Body moult was assessed because juveniles do not moult flight or tail feathers prior to their first autumn migration [32], and body feathers comprise 70% of a bird's total plumage mass [34], [35].

### Assessing condition

Condition (age = 8 d) in the nest was assessed using mass while controlling for tarsus length (structural size). In Savannah sparrows, tarsus length is positively correlated with overall skeletal size [36]. Because body mass may not scale linearly with tarsus length ([37], although see [38]), curvilinear relationships between mass and tarsus were considered in each model. For the 1989–2001 dataset used in the analysis of 1^st^ year survival the relationship between mass and tarsus was slightly convex and so a curvilinear term was included in this model (Fig. S1a). In all other models the relationship was linear (curvilinear terms were not statistically significant), so a linear term for tarsus length was used (Fig. S1b, Fig. S1c, Fig. S2a, Fig. S2b).

Body condition during the pre-migratory period was assessed using (1) total body water, a surrogate for lean tissue mass (muscle+organ mass+gut contents), and (2) fat mass. Both of the latter measures were assessed using heavy water dilution [39]–[41]. For a detailed summary of our methods involving heavy water dilution, please refer to Rae *et al.* [42]. Briefly, heavy water dilution involves introducing a known quantity and concentration of deuterium labeled water (D_2_O) into the body of an organism, allowing it to reach equilibrium, and then taking a blood sample to measure the concentration in the water distilled from the blood. The dilution of D_2_O is then multiplied by the initial injection mass of D_2_O and a correction factor for the exchange of the heavy hydrogen with non-water molecules to estimate the mass of total body water.

Fat mass was measured using the residuals of the regression between body mass and total body water while controlling for tarsus length and repeated measures on individuals through time with a random effect. We measured fat mass using heavy water dilution, because in Savannah sparrows, visual fat scores are not a reliable measure of actual quantities of fat, particularly for low fat scores [43]. Although we lack a validated model for both our measures of total body water and our index of fat mass (see [42]), we suggest both measures of body condition are robust. First, with respect to our estimates of total body water representing an index of lean tissue mass, Karasov and Pinshow [40] found a strong correlation between deuterium dilution space and lean tissue mass as determined via chemical extraction (R^2^ = 0.84) in a similar sized species to the Savannah sparrow, the black cap *Sylvia atricapilla*. Second, any unexplained variation in mass not accounted for by total body water after accounting for skeletal size will be mostly due to fat.

For nestlings of altricial species, such as the Savannah sparrow, measurements of total body water using heavy water dilution may over estimate lean tissue mass for two reasons. First, deuterium may be incorporated into rapidly growing tissues, over estimating deuterium dilution space and total body water [44]. Second, the tissues of young nestling songbirds retain extra water [45]. However, the growth rate of both lean and dry mass in nestling Savannah sparrows reach their asymptotes by approximately 8 d of age [46] and excess tissue water in similarly sized rufous-winged sparrows *Aimophila carpalis* declines to adult levels by approximately 7 d of age [45]. Therefore, given the age of our study individuals during the pre-migratory period (≥27 d of age), heavy water dilution provides an accurate measure of lean tissue mass.

In 2008, 2009, and 2010, three, eight, and 22 individuals, respectively, were carrying radio transmitters during the pre-migratory period (0.62 g; Lotek, Newmarket, Ontario) as part of a larger study on migratory movements. In a recent analysis, we showed that radio transmitters had no effect on total body water or quantity of fat before migration [42]. Also, inclusion of a radio term in our models (0 = no radio, 1 = radio) was not significant and did not change the sign or magnitude of any parameter estimates in our final models (Table S5, Table S6, Table S7). Thus, for parsimony, this term was not included in our final models. Additionally, in 2008, all nestlings from 12 nests received phosphate-buffered saline as a nutritional supplement and all nestlings from four nests received a hypothesized immune system booster (lysozyme). Similarly, in 2009, one nestling from three nests also received phosphate buffered saline. However, neither treatment had an effect on nestling mass (Table S8) and removing these individuals had no effect on the relationship between clutch size and nestling mass in either our observational or experimental analyses (Table S9, Table S10), and so were not censored from any analysis.

### Measuring survival

Individuals were considered to have survived their first year if they were observed in any year following their natal year. Re-sighting probability within the study site was assumed to be close to 100% because observations of breeding behavior and nest monitoring ensured that individuals were sighted almost daily.

### Statistical analyses

All analyses were conducted in R version 2.8.1 using the ‘nlme’ or the ‘MASS’ packages [47]–[49]. The effects of conditions during development on pre-migratory body condition were analyzed in a generalized multi-level path (GMLP) modeling framework using restricted maximum likelihood estimates [50], [51]. A GMLP model is a type of path analysis that can easily accommodate small sample sizes, non-normally distributed data, non-linear functional relationships, and random effects [50], [51]. The fit of a GMLP model is assessed using the concept of d-sep (distance separation) tests [50]. A d-sep test represents a test of the statistical independence between two variables. Shipley [50] shows that for each acyclic path model, there is a subset of independence tests referred to as a “minimum basis set” that account for all possible independence relationships. The null probabilities (P-values) from each test are used to calculate Fisher's C statistic: C = −2∑ln(P)), where P represents the null probability (the P-value) of each d-sep test (total = K), and where C follows a χ^2^ distribution with 2*K degrees of freedom [50], [51]. A significant P-value for this test (P<0.05), suggests the structure of the path model is not consistent with the empirical data. Once model fit is assessed, each path or structural equation is parameterized using the appropriate statistical model.

All model fitting was done using generalized linear mixed effects models. Significance of parameter estimates was evaluated at α = 0.05. Significance of random effects was evaluated using 95% confidence intervals for the SD estimate of the intercepts. Prior to model fitting, potential curvilinear relationships were visually assessed using scatter plots fitted with loess lines. Model fit was visually assessed using residual plots. Correlations between predictors were evaluated through the variance-covariance matrix of the fitted model (r≥0.7); in no case were model terms highly correlated. The approximate proportion of variation accounted for by each model was assessed via regressions of observed and fitted values derived from models without random effects. For the path analysis, each variable was standardized ([value−X̄]/SD) such that path coefficients represent standardized partial regression coefficients, or the standard deviation change in y when x is increased or decreased by one SD [50].

Two-way interactions were initially assessed one at a time using the cross-product values of the terms involved in the interactions (Table S11). For those interactions that were significant, we replaced the cross-product terms with the standardized cross-product residuals according to Lance [52]. This procedure eliminates covariances between the interaction term and the terms involved in the interaction, thereby reducing the number of parameters (paths) in the path model. Only significant interactions were included in the final path model for parsimony.

### Model predictions and hypotheses

#### Brood reduction experiment

We predicted that individuals from reduced broods would be in better condition at day 8 in the nest (see Introduction for hypotheses). We assumed that parental effort did not vary with clutch size [53]. Sample sizes were too small to experimentally assess the effect of number of nestlings on pre-migratory condition (n<10 recaptures of individuals from reduced treatments; Table S4). A random effect for natal nest was included to control for covariance among nest mates (Table S4).

#### Analysis of long-term data: 1^st^ year survival

We predicted nestling mass would positively influence 1^st^ year survival (see Introduction for hypotheses) and that the timing of nesting would negatively influence survival. We made the latter prediction because optimal conditions for migration are hypothesized to decrease through time, possibly limiting survival immediately before or during migration [54], [55]. Random effects for natal nest nested within mother id nested within year were included to control for covariances among nest mates, related individuals, and individuals from the same cohort.

#### Path analysis: breeding period

**Figure 1:**
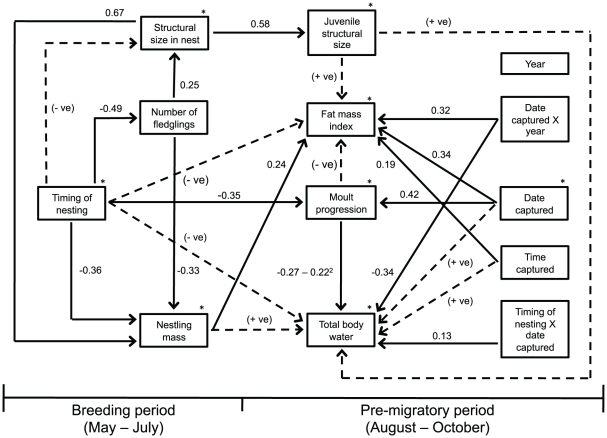
Path model illustrating relationships between natal history and pre-migratory body condition. Solid black arrows represent significant relationships. Numbers associated with each solid line represent standardized path coefficients. The asterisks indicate significant year effects. Hatched lines represent hypothesized causal relationships that were not statistically significant (P>0.05). The sign inside the parentheses associated with each hatched line represents the predicted direction of the relationship.

We predicted that nestling mass would be negatively influenced by the timing of nesting and brood size (Fig. 1; see Introduction for hypotheses). We controlled for year because of potential differences in resource availability, weather, and predators across years [31], [56]. We examined two-way interactions between timing of nesting and year with number of nestlings and nestling mass, as well as two-way interactions between clutch size with year, timing of nesting with nestling mass, and clutch size with nestling mass (Table S11). These interactions were examined because relationships between variables may vary across time owing to potential differences in environmental conditions both within and across years. Again, a random effect for natal nest was included to control for covariances among nest mates. Only one randomly selected nest per adult female was included in the analysis.

#### Path analysis: pre-migratory period

We predicted that nestling mass would be positively correlated with total body water and fat mass because individuals in poor condition may not be able to compensate for their poor start owing to impaired cognition, increased susceptibility to disease both in and out of the nest, or size related dominance hierarchies at high quality foraging locations [25], [57], [58], and that timing of nesting would negatively influence total body water and fat mass, because early fledging birds likely have a head start for accumulating lean tissue and fat mass relative to late fledging individuals. We also predicted that the progression of moult would negatively influence total body water given increased protein demands by tissues that support feather growth [59]–[62], and that moult would negatively influence fat mass given increased thermoregulatory costs and a higher basal metabolic rate during moult ([59], [63]–[67]). We included time captured in our path model because body mass may vary with time of day [59], [68]. We included date captured because migratory birds generally improve condition through time in preparation for migration [26], [69]. A term for year was also included. We examined two-way interactions between nestling mass, timing of nesting, and moult progression with date captured and year, and two-way interactions between nestling mass with timing of nesting and year with date captured (Table S11). Owing to ontogeny, we also examined whether timing of nesting and date of capture influenced moult progression [66], [70]. Last, we tested for differences in moult progression across years and examined the effect of two-way interactions between timing of nesting, date captured, and year on moult (Table S11). All structural equations for the pre-migratory period included a random effect for individual nested within natal nest to control for covariances within individuals and among nest mates, respectively.

### Ethical treatment of animals

Research was carried out in strict accordance with the recommendations in the Guidelines to the Use of Wild Birds in Research (Available: http://www.nmnh.si.edu/BIRDNET). This study was approved by the University of Guelph Animal Care Committee (Permit Number: R/A 08R061), the Bowdoin College Research Oversight Committee (Permit Number: 2009-18 r2011), and the Canadian Wildlife Service (Scientific Collection Permit Number: SC2732, Banding Permit Number: 10789D).

## Results

### Brood reduction experiment

**Figure 2:**
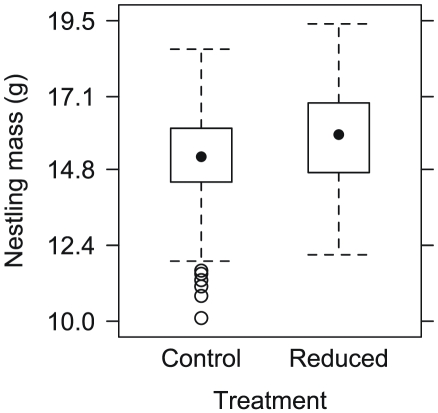
Box plot illustrating lower nestling mass (g) for control broods (4 fledglings) relative to experimentally reduced broods (3 fledglings). Solid circles in the middle of each box represent median values, the boxes encompass the inter-quantile range (25–75%), and the whiskers and hollow points represent the outer 25% quantiles.

Nestling mass was significantly higher in the reduced treatment compared with the control group (β = 1.20, t = 3.7, df = 35, P = 0.001, Fig. 2), and varied positively with tarsus length (β = 1.04, t = 11.5, df = 95, P<0.001). We did not find evidence for a relationship between nestling mass with either timing of nesting (β = −0.01, t = −1.0, df = 35, P = 0.321) or year (2010: β = 0.16, t = 0.51, df = 35, P = 0.616).

### Analysis of long-term data: 1^st^ year survival

**Figure 3:**
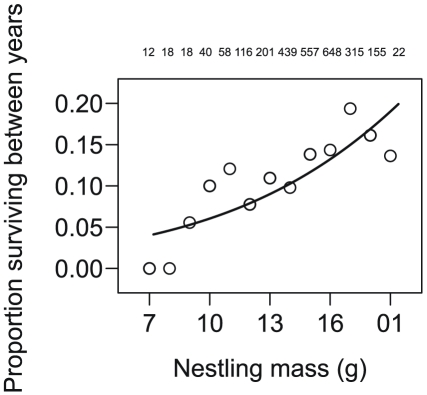
Scatter plot illustrating positive relationship between nestling mass and 1^st^ year survival. Numbers above points represent sample sizes for each weight class. Weight classes represent integer values of original weights. Trend illustrated with fitted values from a logistic regression model detailing the relationship between nestling mass and survival after controlling for covariances among related individuals (mother ID), nest mates (natal nest), and cohorts (year).

First-year survival was positively related to nestling mass (β = 0.13, t = 3.2, df = 1889, P = 0.001, Fig. 3), negatively related to the timing of nesting (β = −0.01, t = −2.3, df = 114, P = 0.021), and positively related to tarsus length (linear term for tarsus length: β = 2.27, *t* = 2.4, df = 1889, *P* = 0.018; curvilinear term for tarsus length: β = −0.06, *t* = −2.4, df = 1889, *P* = 0.017). Regressing the logit from the fitted model on the observed binomial response indicated that our model explained 24% of the observed variation in the probability of survival.

### Path analysis: breeding period

Our hypothesized path model (Fig. 1) was consistent with the structure of our data (Fisher's C statistic = 119.32, df = 100, P = 0.091, i.e., our proposed model did not significantly differ from the correlational structure of the data). Timing of nesting was significantly later in 2009 than in 2008, but did not vary between 2008 and 2010 (2009: β = 0.90, t = 2.5, df = 57, P = 0.015; 2010: β = 0.22, t = 0.7, df = 57, P = 0.469; Fig. 1). As predicted, the number of fledglings decreased as timing of nesting increased (β = −0.49, t = −4.0, df = 56, P<0.001), but we did not find any evidence for a difference between years (2009: β = 0.01, t = 0.2, df = 56, P = 0.986; 2010: β = 0.35, t = 1.3, df = 56, P = 0.207). The main effects in our structural equation accounted for approximately 23% of the observed variation in number of fledglings.

**Figure 4:**
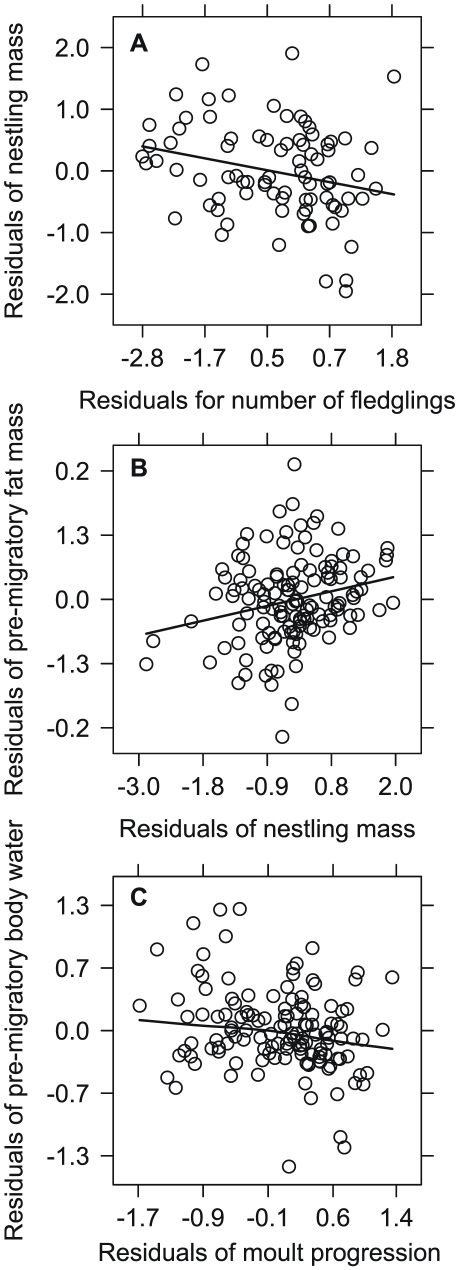
Partial regression plots depicting factors affecting body condition. Panels illustrate: (A) negative relationship between nestling mass with number of fledglings, (B) positive relationship between fat mass and nestling mass, and (C) negative curvilinear relationship between total body water and moult progression. Trends illustrated with locally weighted regression lines.

As predicted, nestling mass decreased as both the number of fledglings (β = −0.33, t = −3.6, df = 55, P = 0.001; Fig. 1, Fig. 4a, Table S12) and timing of nesting (β = −0.36, t = −3.8, df = 55, P<0.001) increased. Nestling mass also varied positively with tarsus length (β = 0.67, t = 8.9, df = 51, P<0.001) and was greater in 2010 compared with 2008 (β = 0.43, t = 2.2, df = 55, P = 0.031), however, there was little evidence for a difference between 2008 and 2009 (β = 0.34, t = 1.4, df = 55, P = 0.174). Tarsus length increased as the number of nestlings increased (β = 0.25, t = 2.2, df = 55, P = 0.035) and was larger in both 2009 and 2010 relative to 2008 (2009: β = 0.66, t = 2.1, df = 55, P = 0.038; 2010: β = 0.61, t = 2.6, df = 55, P = 0.014). We did not find any evidence for a relationship between tarsus length and timing of nesting (β = −0.08, t = −0.7, df = 55, P = 0.509). The main effects in our structural equations accounted for approximately 53% of the observed variation in fledging mass and 14% of the observed variation in tarsus length.

### Path analysis: pre-migratory period

The average number of captures during the pre-migratory period per individual was 4.8 in 2008 (range = 2–11, SD = 2.2, n = 33), 3.7 in 2009 (range = 2–8, SD = 1.4, n = 22), and 3.5 in 2010 (range = 2–11, SD = 1.63, n = 44). Average age at capture was 59, 66, and 63 d in 2008, 2009, and 2010, respectively (2008: range = 27–114, SD = 21.7, n = 52; 2009: range = 48–105, SD = 15.2, n = 36; 2010: range = 32–98, SD = 15.2, n = 81).

As predicted, we found a positive relationship between fat mass during the pre-migratory period and nestling mass (β = 0.24, t = 2.9, df = 28, P = 0.008; Fig. 1, Fig. 4b, Table S12), but only weak evidence for a relationship between total body water and nestling mass (β = 0.16, t = 1.9, df = 28, P = 0.064). Also as predicted, moult progression influenced total body water (linear term for moult progression: β = −0.27, *t* = −2.61, df = 48, *P* = 0.012; curvilinear term for moult progression: β = −0.22, t = −2.33, df = 48, P = 0.024; Fig. 4c), but we did not find evidence for a relationship between fat mass and moult progression (β = 0.13, t = 1.2, df = 50, P = 0.219). Total body water varied with timing of nesting, however, this relationship was dependent on date captured: total body water did not vary with date captured for birds fledging early or in the middle of the breeding period, but increased as date captured increased for late fledging birds (β = 0.13, t = 2.1, df = 48, P = 0.040; Fig. S3, Table S11). We did not find any evidence for a relationship between pre-migratory fat mass and timing of nesting (β = −0.08, t = −0.9, df = 46, P = 0.399).

Both total body water and fat mass varied with date captured, but the direction of these relationships was different across years. Specifically, total body water increased as date captured increased in 2008, but decreased during 2009 and 2010 (β = −0.34, t = −5.8, df = 48, P<0.001; Fig. S4a–c). Conversely, fat mass did not change with date captured in 2008, but increased with date captured in 2009 and 2010 (β = 0.32, t = 4.3, df = 50, P<0.001; Fig. S4d–f). After controlling for these interactions, total body water was higher on average in 2009 relative to 2008 (β = 1.23, t = 4.8, df = 46, P<0.001), but we did not find evidence for a difference between 2008 and 2010 (β = −0.21, t = −1.0, df = 46, P = 0.329). Conversely, fat mass was on average lower in 2009 relative to 2008 (β = −0.68, t = −2.9, df = 46, P = 0.006), but again, we did not find evidence for a difference between 2008 and 2010 (β = −0.38, t = −1.8, df = 46, P = 0.077). Fat mass increased with time of day (time captured: β = 0.19, t = 2.6, df = 50, P = 0.014; Fig. 1, Table S12), but we did not find evidence for a similar relationship with total body water (β = 0.07, t = 1.2, df = 48, P = 0.252). We also did not find any evidence for a relationship between total body water or fat mass with tarsus length (total body water: β = 0.12, t = 1.4, df = 28, P = 0.183; fat mass: β = −0.11, t = −1.3, df = 28, P = 0.210). The main and interaction effects in our structural equations for total body water and fat mass accounted for approximately 54% and 36% of the observed variation in each variable, respectively.

As predicted, the progression of body moult was negatively influenced by the timing of nesting (β = −0.35, t = −4.0, df = 46, P<0.001) and increased as date captured increased (β = 0.42, t = 7.0, df = 53, P<0.001). Progression of body moult was also more advanced in 2010 than in 2008, but we did not find any evidence for a difference between 2008 and 2009 (2009: β = 0.26, t = 1.0, df = 46, P = 0.302; 2010: β = 1.03, t = 5.1, df = 46, P<0.001). The main effects in this structural equation accounted for approximately 50% of the observed variation in moult progression. As expected, tarsus length was strongly and positively related to tarsus length in the nest (β = 0.58, t = 7.5, df = 50, P<0.001).

## Discussion

Our study is the first to simultaneously show that nestling mass is affected by brood size (through our brood manipulation experiments), that fledging condition carries over to affect pre-migratory fat stores immediately prior to migration, and that nestling condition is linked with 1^st^ year survival in the same migratory population. A key prediction of the hypothesis that conditions during development limit survival during migration through their effects on pre-migratory body condition is demonstration of a positive relationship between nestling mass and pre-migratory body condition. Although we did not measure fat stores in nestling Savannah sparrows, recent evidence suggests that differences in nestling mass may be attributable to differences in quantities of fat [71], [72], further suggesting that the positive relationship we observed between nesting mass and pre-migratory fat mass represents a positive correlation between fat stores in the nest and fat stores prior to migration. This correlation may be maintained by several mechanisms. For example, birds in poor body condition may be more susceptible to disease [73], [74], resulting in additional maintenance costs that limit fat accumulation [25], [75]. Alternatively, nutritional stress during development may impair cognitive function [57], affecting an individual's ability forage efficiently and accumulate fat. Individuals fledging in better condition may also exclude individuals in poor condition from favorable foraging sites [25], [58], [76], again preventing individuals from compensating for their poor condition. Further studies linking conditions during development with body condition, behavior, and immune system function during the pre-migratory period are needed to adequately test these hypotheses.

Indirect effects in path models are calculated as the product of the path coefficients along the indirect path (Fig. 1). Our path model suggests that timing of nesting may affect pre-migratory body condition through two indirect mechanisms. First, timing of nesting had a negative indirect effect on pre-migratory fat mass through its effect on nestling mass (−0.33×0.24 = −0.08). Second, the negative effect of timing of nesting on moult progression (see also [66], [70]), and the negative effect of moult progression on total body water (see also [59]–[62]), results in a positive indirect effect of timing of nesting on total body water (−0.35×(−0.27–0.22^2^) = 0.09+0.07^2^). However, this ‘positive’ effect results from late fledging individuals having yet to incur the lean tissue costs of moult. Perhaps this impending cost explains why we observed late fledging birds increasing lean tissue mass throughout the pre-migratory period, but did not see the same pattern for earlier fledging birds; late fledging birds may have been preemptively trying to mitigate the costs of moult associated with timing of nesting.

Moult requires substantial amounts of protein to replace the integument that supports feather growth [60], [61]. However, most studies investigating the costs of moult in songbirds have taken place in the laboratory, where food is available *ad libitum* (e.g., [60], [61]), making it difficult to assess how moult may affect lean tissue mass under natural conditions. Furthermore, when protein intake has been restricted, it is often at levels far below what would be encountered in the wild, and control groups are often still fed *ad libitum* (e.g., [77]). Our results suggest that moult progression may deplete pre-migratory lean tissue mass in wild birds. Similarly, Baggott [59] found that pectoralis muscle mass was negatively correlated with moult progression in free-living willow warblers *Phylloscopus trochilus*, and Bauchinger and Biebach [62] found that kidney and pancreas mass was negatively correlated with moult progression in garden warblers *Sylvia borin*. Our results and those of these other studies suggest the importance of assessing lean tissue costs of moult under natural conditions.

Our results suggest that the timing of nesting influences moult progression. Similar results have also been found for juvenile great tits *Parus major* and Savi's warblers *Locustella luscinioides* [66], [70]. This pathway represents another mechanism through which early life events may limit survival during migration, or even during the subsequent wintering period. For example, the rate of moult (not assessed here) may increase for late fledging individuals, resulting in lower quality plumage, which in turn may result in decreased flight performance and impaired thermoregulation [78]–[80]. Alternatively, delays in moult could constrain the timing of migratory departure [81]. To address these hypotheses in migratory species, improvements in tracking technologies are needed to follow individuals from birth and monitor subsequent survival rates at each stage of the annual cycle.

We found that pre-migratory total body water increased with date captured in 2008, but not in 2009 or 2010. Conversely, pre-migratory fat mass did not increase with date captured in 2008, but increased in both 2009 and 2010. These results suggest that individuals may be choosing to invest in either lean tissue mass or fat mass depending on current or anticipated conditions. For example, when food availability is unpredictable, songbirds tend to invest in fat mass [82], [83]. Alternatively, annual differences in food availability or weather conditions may constrain what aspects of body condition are to be invested towards. In our study region, the average daily precipitation for the months of August and September were 4.0, 2.8, and 2.3 mm in 2008, 2009, and 2010 respectively (www.climate.weatheroffice.gc.ca). Juvenile songbirds are inefficient foragers, and rainstorms are expected to hamper their foraging abilities even further [84]. Therefore, the ‘unpredictable foraging conditions’ hypothesis predicts that individuals should invest in fat mass during 2008, however this is opposite to what we observe. Instead, increasing lean tissue mass in 2008 appears to be linked to precipitation through some other (unknown) causal mechanism.

Our results indicate that nestlings from larger broods had lower mass (controlling for tarsus length). However, opposite to our prediction, nestlings from larger broods had longer tarsi, suggesting that tarsus length in the nest is not affected by the same nutritional or energetic mechanisms as mass. Structural traits are often heritable in songbirds [85], [86], and breeding females with longer tarsi tend to be high quality individuals that produce larger clutches [85]. Therefore, the observed positive relationship between clutch size and tarsus length may be caused by a correlation between clutch size and tarsus length in the mother. Regardless of the exact mechanism resulting in this correlation, the net effect of clutch size on nestling mass was still negative (Fig. 1; sum of direct and indirect effects of clutch size on nestling mass = (0.25×0.67)+−0.33 = −0.16).

Migration is energetically and physically demanding; lean tissue mass and fat mass are both critical to a successful migration [26], [27]. Here we provide evidence that conditions during development affect survival during migration through their effects on pre-migratory body condition. Understanding these relationships is important given recent evidence that climate change and anthropogenic changes to landscape structure may affect the growth and development of nestlings (e.g., [87], [88]). Future research investigating the effects of environmental perturbations on migratory species should consider how early developmental conditions carry-over to influence individual success in subsequent life-history stages.

## Supporting Information

Figure S1 **Scatterplots illustrating relationships between mass (g) and tarsus length (mm).** Panels represent: (A) 1989–2001 dataset used to examine the relationship between 1^st^ year survival and nestling mass, (B) 2008–2010 dataset examining the factors affecting nestling mass, and (C) 2008–2010 dataset used to derive the fat mass index. Trends illustrated with locally weighted regression lines.
(EPS)

Figure S2 **Scatterplots illustrating relationships between pre-migratory body condition and tarsus length.** Panels represent: (A) pre-migratory total body water (g) and tarsus length (mm) and (B) pre-migratory fat mass and tarsus length (mm). Trends illustrated with locally weighted regression lines.
(EPS)

Figure S3 **Partial regression plots for total body water, illustrating the interaction between date captured and timing of nesting.** Panels represent: (A) birds fledging early in the breeding period, (B) birds fledging in the middle of the breeding period, and (C) birds fledging late in the breeding period. This figure suggests that late fledging individuals may increase lean tissue mass rapidly to catch-up with early fledging conspecifics. Date captured and timing of nesting were not included in models generating residuals for the plots. Trends illustrated with locally weighted regression lines.
(EPS)

Figure S4 **Partial regression plots illustrating the interaction between date captured and year for both total body water (A–C) and fat mass (D–F).** Panels represent: (A) positive relationship between total body water and date captured for 2008, (B, C) shallow negative relationships between total body water and date captured for 2009 and 2010, respectively, (D) very shallow negative relationship between fat mass and date captured for 2008, (E, F) positive relationships between fat mass and date captured in 2009 and 2010, respectively. This figure suggests investment strategies in lean tissue mass or fat mass may vary depending on either current or anticipated environmental conditions. Date captured and year, were not included in models generating residuals for the plots. Trends illustrated with locally weighted regression lines.
(EPS)

Table S1 **Factors affecting nestling mass for datasets both (1) including and (2) excluding nestlings (n = 3) for which age was estimated.** A random effect was included for natal nest. Reference level for year is 2008. Parameter estimates based on standardized data.
(DOC)

Table S2 **Factors affecting 1^st^ year survival for datasets both (1) including and (2) excluding nestlings (n = 188) for which age was estimated using plumage characteristics and behavior.** Random effects were included for natal nest, nested in mother, nested in year. ^2^ indicates curvilinear term. Parameter estimates based on un-standardized data.
(DOC)

Table S3 **Model results from experimental brood manipulations carried out in 2009.** Both brood enlargements (n = 29 nestlings from n = 6 nests) and reductions (n = 26 nestlings from n = 9 nests) were carried out. One nestling from each treatment group was removed because tarsus length measurements were not obtained. Control nests were comprised of four nestlings. A random effect was included for natal nest. Parameter estimates based on un-standardized data.
(DOC)

Table S4 **Sample sizes for each analysis by year.** “Reduced” and “Control” refer to experimental brood reductions (3 nestlings) and controls (4 nestlings), respectively. Numbers in parentheses represent original sample sizes prior to removing non-independent samples (see Methods: Model predictions and hypotheses).
(DOC)

Table S5 **Factors affecting pre-migratory fat mass when a term for radio transmitter is (1) excluded and (2) included (n = 33).** Random effects were included for individual nested within natal nest. Reference level for year is 2008. Parameter estimates based on standardized data.
(DOC)

Table S6 **Factors affecting pre-migratory moult progression when a term for radio transmitter is (1) excluded and (2) included (n = 33).** Random effects were included for individual nested within natal nest. Reference level for year is 2008. Parameter estimates based on standardized data.
(DOC)

Table S7 **Factors affecting pre-migratory total body water when a term for radio transmitter is (1) excluded and (2) included (n = 33).** Random effects were included for individual nested within natal nest. ^2^ indicates curvilinear term. Reference level for year is 2008. Parameter estimates based on standardized data.
(DOC)

Table S8 **Model results for supplementation experiments carried out in (1) 2008 and (2) 2009.** Nestlings were provided with a hypothesized immune system booster (lysozyme) or a hypothesized nutritional supplement (phosphate-buffered saline; PBS) every other day prior to day 8 (2008: n = 70 for PBS, n = 33 for lysozyme; 2009: n = 59 for PBS). Lysozyme was not used in 2009. Controls represent un-manipulated individuals. A random effect was included for natal nest. Experiments carried out by RA Mauck. Parameter estimates based on un-standardized data.
(DOC)

Table S9 **Factors affecting nestling mass for datasets both (1) including and (2) excluding nestlings that received phosphate-buffered saline (PBS) or lysozyme.** Sample sizes in 2008: n = 27 from n = 12 nests for PBS, n = 14 from 4 nests for lysozyme. Sample sizes in 2009: n = 3 from n = 3 nests for PBS. A random effect was included for natal nest. Reference level for year is 2008. Parameter estimates based on standardized data.
(DOC)

Table S10 **Results from experimental brood manipulations when nestling that received PBS are (1) included and (2) excluded (n = 5).** A random effect was included for natal nest. Reference level for year is 2009. Parameter estimates based on un-standardized data.
(DOC)

Table S11 **Model results (interactions only; no main effects) for hypothesized two-way interactions.** ‘X’ denotes an interaction. The moult progression X year interaction for fat mass was no longer significant after the other interactions were removed from the model (year: 2009: β = −0.54, t = −1.83, DF = 47, P = 0.07; year: 2010: β = −0.02, t = −0.11, DF = 47, P = 0.91). Also, because the effect size and significance of interaction terms for date captured and year were similar in 2009 and 2010 for both total body water and fat mass, year was recoded as ‘2008’ and ‘2009+2010’ in order to reduce the number of parameters and paths included in the final path model. Random effects were included for individual nested within natal nest. Parameter estimates based on standardized data.
(DOC)

Table S12 **Factors affecting: (1) Nestling mass, (2) pre-migratory total body water, (3) pre-migratory fat mass, and (4) pre-migratory moult progression.** For number 1, a random effect was included for natal nest. For numbers 2 through 4, random effects were included for individual nested within natal nest. Reference level for year is 2008. ^2^ represents curvilinear term and ‘X’ denotes an interaction. Parameter estimates based on un-standardized data.
(DOC)
