# Supplementary material for: Early Life Events Carry Over to Influence Pre-Migratory Condition in a Free-Living Songbird
Source: PLoS One. 2011 Dec 16;6(12):e28838. doi: 10.1371/journal.pone.0028838 (PMC3241683; doi:10.1371/journal.pone.0028838)
Supplement: Table S1 — Factors affecting nestling mass for datasets both (1) including and (2) excluding nestlings (n = 3) for which age was estimated. A random effect was included for natal nest. Reference level for year is 2008. Parameter estimates based on standardized data. (DOC) [file pone.0028838.s005.doc]

| **Model** | **Model Term** | **β** | **t** | **df** | **P (t)** |
| --- | --- | --- | --- | --- | --- |
| (1) Dataset including individuals for which age in nest estimated | Timing of nesting | -0.36 | -3.83 | 55 | <0.001 |
|  | Number of fledglings | -0.33 | -3.56 | 55 | <0.001 |
|  | Tarsus length | 0.67 | 8.93 | 51 | <0.001 |
|  | Year: 2009 | 0.34 | 1.38 | 55 | 0.174 |
|  | Year: 2010 | 0.43 | 2.21 | 55 | 0.032 |
| (2) Dataset excluding individuals for which age in nest estimated | Timing of nesting | -0.40 | -3.81 | 52 | <0.001 |
|  | Number of fledglings | -0.35 | -3.64 | 52 | <0.001 |
|  | Tarsus length | 0.67 | 8.81 | 50 | <0.001 |
|  | Year: 2009 | 0.38 | 1.49 | 52 | 0.143 |
|  | Year: 2010 | 0.48 | 2.37 | 52 | 0.022 |
