# Supplementary material for: Early Life Events Carry Over to Influence Pre-Migratory Condition in a Free-Living Songbird
Source: PLoS One. 2011 Dec 16;6(12):e28838. doi: 10.1371/journal.pone.0028838 (PMC3241683; doi:10.1371/journal.pone.0028838)
Supplement: Table S2 — Factors affecting 1st year survival for datasets both (1) including and (2) excluding nestlings (n = 188) for which age was estimated using plumage characteristics and behavior. Random effects were included for natal nest, nested in mother, nested in year. 2 indicates curvilinear term. Parameter estimates based on un-standardized data. (DOC) [file pone.0028838.s006.doc]

| **Model** | **Model Term** | **β** | **t** | **df** | **P (t)** |
| --- | --- | --- | --- | --- | --- |
| (1) Dataset including individuals for which age was estimated | Nestling mass | 0.13 | 3.21 | 1889 | 0.001 |
|  | Tarsus | 2.27 | 2.37 | 1889 | 0.018 |
|  | Tarsus^2^ | -0.06 | -2.39 | 1889 | 0.017 |
|  | Timing of nesting | -0.01 | -2.35 | 114 | 0.021 |
| (2) Dataset excluding individuals for which age was estimated | Nestling mass | 0.14 | 2.79 | 1214 | 0.005 |
|  | Tarsus | 1.74 | 1.48 | 1214 | 0.140 |
|  | Tarsus^2^ | -0.04 | -1.49 | 1214 | 0.136 |
|  | Timing of nesting | -0.02 | -2.93 | 46 | 0.005 |
