# Supplementary material for: Early Life Events Carry Over to Influence Pre-Migratory Condition in a Free-Living Songbird
Source: PLoS One. 2011 Dec 16;6(12):e28838. doi: 10.1371/journal.pone.0028838 (PMC3241683; doi:10.1371/journal.pone.0028838)
Supplement: Table S3 — Model results from experimental brood manipulations carried out in 2009. Both brood enlargements (n = 29 nestlings from n = 6 nests) and reductions (n = 26 nestlings from n = 9 nests) were carried out. One nestling from each treatment group was removed because tarsus length measurements were not obtained. Control nests were comprised of four nestlings. A random effect was included for natal nest. Parameter estimates based on un-standardized data. (DOC) [file pone.0028838.s007.doc]

| **Model Term** | **β** | **t** | **df** | **P (t)** |
| --- | --- | --- | --- | --- |
| Enlarged broods (5 nestlings) | 0.29 | 0.58 | 22 | 0.568 |
| Reduced broods (3 nestlings) | 1.06 | 2.32 | 22 | 0.030 |
| Timing of nesting | -0.02 | -1.44 | 22 | 0.163 |
| Tarsus length | 1.39 | 16.32 | 72 | <0.001 |
