# Supplementary material for: Early Life Events Carry Over to Influence Pre-Migratory Condition in a Free-Living Songbird
Source: PLoS One. 2011 Dec 16;6(12):e28838. doi: 10.1371/journal.pone.0028838 (PMC3241683; doi:10.1371/journal.pone.0028838)
Supplement: Table S4 — Sample sizes for each analysis by year. “Reduced” and “Control” refer to experimental brood reductions (3 nestlings) and controls (4 nestlings), respectively. Numbers in parentheses represent original sample sizes prior to removing non-independent samples (see Methods: Model predictions and hypotheses). (DOC) [file pone.0028838.s008.doc]

| **Analysis** | **Year** | **n nests** | **n individuals** | **n captures** |
| --- | --- | --- | --- | --- |
| Path analysis:  Breeding period | 2008 | 22 (32) | 51 (66) | NA |
|  | 2009 | 13 (17) | 18 (24) | NA |
|  | 2010 | 25 (30) | 43 (54) | NA |
| Path analysis:  Pre-migratory period | 2008 | 19 (23) | 27 (33) | 42 (52) |
|  | 2009 | 12 (16) | 17 (22) | 29 (38) |
|  | 2010 | 19 (22) | 36 (44) | 63 (81) |
| Brood reductions: Breeding period | 2008 | NA | NA | NA |
|  | 2009 | Reduced: 9 (9)  Control: 11 (12) | Reduced: 26 (26)  Control: 41 (48) | NA |
|  | 2010 | Reduced: 6 (7) Control: 14 (22) | Reduced: 17 (20)  Control: 55 (87) | NA |
| Brood reduction:  Pre-migratory Period | 2008 | NA | NA | NA |
|  | 2009 | Reduced: 3 (3)  Control: 4 (4) | Reduced: 3 (5)  Control: 4 (6) | Reduced: 5 (6)  Control: 11 (15) |
|  | 2010 | Reduced: 5 (5)  Control: 6 (11) | Reduced: 6 (7)  Control: 5 (21) | Reduced: 7 (10)  Control: 11 (38) |
| Long-term dataset:  First-year survival | 1989 | 70 | 253 | NA |
|  | 1990 | 54 | 197 | NA |
|  | 1991 | 63 | 216 | NA |
|  | 1992 | 66 | 238 | NA |
|  | 1993 | 57 | 230 | NA |
|  | 1994 | 54 | 205 | NA |
|  | 1995 | 55 | 198 | NA |
|  | 1996 | 36 | 145 | NA |
|  | 1997 | 43 | 157 | NA |
|  | 1998 | 43 | 169 | NA |
|  | 1999 | 51 | 195 | NA |
|  | 2001 | 57 | 205 | NA |
