# Supplementary material for: Early Life Events Carry Over to Influence Pre-Migratory Condition in a Free-Living Songbird
Source: PLoS One. 2011 Dec 16;6(12):e28838. doi: 10.1371/journal.pone.0028838 (PMC3241683; doi:10.1371/journal.pone.0028838)
Supplement: Table S5 — Factors affecting pre-migratory fat mass when a term for radio transmitter is (1) excluded and (2) included (n = 33). Random effects were included for individual nested within natal nest. Reference level for year is 2008. Parameter estimates based on standardized data. (DOC) [file pone.0028838.s009.doc]

| **Model** | **Model Term** | **β** | **t** | **df** | **P (t)** |
| --- | --- | --- | --- | --- | --- |
| (1) Model excluding radio transmitter term | Nestling mass | 0.24 | 2.86 | 28 | 0.008 |
|  | Timing of nesting | -0.07 | -0.85 | 46 | 0.398 |
|  | Moult progression | 0.13 | 1.24 | 50 | 0.219 |
|  | Tarsus length | -0.11 | -1.28 | 28 | 0.210 |
|  | Time of day captured | 0.19 | 2.55 | 50 | 0.014 |
|  | Date captured | 0.34 | 3.78 | 50 | <0.001 |
|  | Year: 2009 | -0.67 | -2.88 | 46 | 0.006 |
|  | Year: 2010 | -0.38 | -1.81 | 46 | 0.077 |
|  | Date captured X year | 0.32 | 4.29 | 50 | <0.001 |
| (2) Model including radio transmitter term | Nestling mass | 0.24 | 2.09 | 28 | 0.042 |
|  | Timing of nesting | -0.08 | -0.84 | 46 | 0.407 |
|  | Moult progression | 0.13 | 1.27 | 49 | 0.212 |
|  | Tarsus length | -0.11 | -1.26 | 28 | 0.220 |
|  | Time of day captured | 0.19 | 2.45 | 49 | 0.020 |
|  | Date captured | 0.29 | 3.17 | 50 | <0.001 |
|  | Year: 2009 | -0.71 | -2.91 | 46 | 0.006 |
|  | Year: 2010 | -0.42 | -1.88 | 46 | 0.067 |
|  | Radio transmitter | 0.09 | 0.52 | 49 | 0.608 |
|  | Date captured X year | 0.32 | 4.22 | 49 | <0.001 |
