# Supplementary material for: Early Life Events Carry Over to Influence Pre-Migratory Condition in a Free-Living Songbird
Source: PLoS One. 2011 Dec 16;6(12):e28838. doi: 10.1371/journal.pone.0028838 (PMC3241683; doi:10.1371/journal.pone.0028838)
Supplement: Table S6 — Factors affecting pre-migratory moult progression when a term for radio transmitter is (1) excluded and (2) included (n = 33). Random effects were included for individual nested within natal nest. Reference level for year is 2008. Parameter estimates based on standardized data. (DOC) [file pone.0028838.s010.doc]

| **Model** | **Model Term** | **β** | **t** | **df** | **P (t)** |
| --- | --- | --- | --- | --- | --- |
| (1) Model excluding radio transmitter term | Timing of nesting | -0.35 | -4.02 | 46 | <0.001 |
|  | Date captured | 0.42 | 6.97 | 53 | <0.001 |
|  | Year: 2009 | 0.26 | 1.04 | 46 | 0.302 |
|  | Year: 2010 | 1.03 | 5.08 | 46 | <0.001 |
| (2) Model including radio transmitter term | Timing of nesting | -0.35 | -3.96 | 46 | <0.001 |
|  | Date captured | 0.43 | 7.04 | 52 | <0.001 |
|  | Year: 2009 | 0.29 | 1.15 | 46 | 0.254 |
|  | Year: 2010 | 1.07 | 5.16 | 46 | <0.001 |
|  | Radio transmitter | -0.14 | -1.13 | 52 | 0.260 |
