# Supplementary material for: Early Life Events Carry Over to Influence Pre-Migratory Condition in a Free-Living Songbird
Source: PLoS One. 2011 Dec 16;6(12):e28838. doi: 10.1371/journal.pone.0028838 (PMC3241683; doi:10.1371/journal.pone.0028838)
Supplement: Table S7 — Factors affecting pre-migratory total body water when a term for radio transmitter is (1) excluded and (2) included (n = 33). Random effects were included for individual nested within natal nest. 2 indicates curvilinear term. Reference level for year is 2008. Parameter estimates based on standardized data. (DOC) [file pone.0028838.s011.doc]

| **Model** | **Model Term** | **β** | **t** | **df** | **P (t)** |
| --- | --- | --- | --- | --- | --- |
| (1) Model excluding radio transmitter term | Nestling mass | 0.16 | 1.93 | 28 | 0.060 |
|  | Timing of nesting | 0.06 | 0.67 | 46 | 0.675 |
|  | Moult progression | -0.27 | -2.61 | 48 | 0.012 |
|  | Moult progression^2^ | -0.22 | -2.33 | 48 | 0.024 |
|  | Tarsus length | 0.12 | 1.37 | 28 | 0.183 |
|  | Time of day captured | 0.07 | 1.16 | 48 | 0.252 |
|  | Date captured | 0.14 | 1.96 | 48 | 0.056 |
|  | Year: 2009 | 1.23 | 4.82 | 46 | 0.001 |
|  | Year: 2010 | -0.21 | -0.99 | 46 | 0.329 |
|  | Timing of nesting X date captured | 0.12 | 2.11 | 48 | 0.040 |
|  | Date captured X year | -0.34 | -5.83 | 48 | <0.001 |
| (2) Model including radio transmitter term | Nestling mass | 0.16 | 1.93 | 28 | 0.064 |
|  | Timing of nesting | 0.06 | 0.67 | 46 | 0.505 |
|  | Moult progression | -0.27 | -2.60 | 49 | 0.013 |
|  | Moult progression^2^ | -0.22 | -2.32 | 47 | 0.025 |
|  | Tarsus length | 0.12 | 1.36 | 28 | 0.185 |
|  | Time of day captured | 0.07 | 1.15 | 47 | 0.254 |
|  | Date captured | 0.14 | 1.94 | 47 | 0.058 |
|  | Year: 2009 | 1.29 | 4.77 | 46 | <0.001 |
|  | Year: 2010 | -0.20 | -0.94 | 46 | 0.351 |
|  | Timing of nesting X date captured | 0.13 | 2.07 | 47 | 0.044 |
|  | Date captured X year | -0.34 | -5.79 | 47 | <0.001 |
|  | Radio transmitter | -0.01 | -0.09 | 47 | 0.925 |
