# Supplementary material for: Early Life Events Carry Over to Influence Pre-Migratory Condition in a Free-Living Songbird
Source: PLoS One. 2011 Dec 16;6(12):e28838. doi: 10.1371/journal.pone.0028838 (PMC3241683; doi:10.1371/journal.pone.0028838)
Supplement: Table S8 — Model results for supplementation experiments carried out in (1) 2008 and (2) 2009. Nestlings were provided with a hypothesized immune system booster (lysozyme) or a hypothesized nutritional supplement (phosphate-buffered saline; PBS) every other day prior to day 8 (2008: n = 70 for PBS, n = 33 for lysozyme; 2009: n = 59 for PBS). Lysozyme was not used in 2009. Controls represent un-manipulated individuals. A random effect was included for natal nest. Experiments carried out by RA Mauck. Parameter estimates based on un-standardized data. (DOC) [file pone.0028838.s012.doc]

| **Model** | **Model Term** | **β** | **t** | **df** | **P (t)** |
| --- | --- | --- | --- | --- | --- |
| (1) 2008 experiments | Lysozyme treatment | -0.11 | -0.30 | 107 | 0.760 |
|  | PBS treatment | 0.30 | -0.94 | 107 | 0.346 |
|  | Timing of nesting | -0.03 | -0.03 | 42 | 0.004 |
|  | Number of fledglings | -0.88 | -4.34 | 42 | <0.001 |
|  | Tarsus length | 1.15 | 14.35 | 107 | <0.001 |
| (2) 2009 experiment | PBS treatment | 0.27 | 0.96 | 69 | 0.340 |
|  | Timing of nesting | -0.01 | -0.75 | 28 | 0.457 |
|  | Number of fledglings | -0.87 | -3.28 | 28 | 0.003 |
|  | Tarsus length | 1.28 | 14.96 | 69 | <0.001 |
