# Supplementary material for: Early Life Events Carry Over to Influence Pre-Migratory Condition in a Free-Living Songbird
Source: PLoS One. 2011 Dec 16;6(12):e28838. doi: 10.1371/journal.pone.0028838 (PMC3241683; doi:10.1371/journal.pone.0028838)
Supplement: Table S9 — Factors affecting nestling mass for datasets both (1) including and (2) excluding nestlings that received phosphate-buffered saline (PBS) or lysozyme. Sample sizes in 2008: n = 27 from n = 12 nests for PBS, n = 14 from 4 nests for lysozyme. Sample sizes in 2009: n = 3 from n = 3 nests for PBS. A random effect was included for natal nest. Reference level for year is 2008. Parameter estimates based on standardized data. (DOC) [file pone.0028838.s013.doc]

| **Model** | **Model Term** | **β** | **t** | **df** | **P (t)** |
| --- | --- | --- | --- | --- | --- |
| (1) Dataset including nestlings that received PBS and lysozyme | Timing of nesting | -0.36 | -3.83 | 55 | <0.001 |
|  | Number of fledglings | -0.33 | -3.56 | 55 | <0.001 |
|  | Tarsus length | 0.67 | 8.93 | 51 | <0.001 |
|  | Year: 2009 | 0.34 | 1.37 | 55 | 0.174 |
|  | Year: 2010 | 0.43 | 2.21 | 55 | 0.032 |
| (2) Dataset excluding nestlings that received PBS and lysozyme | Timing of nesting | -0.35 | -3.04 | 37 | 0.004 |
|  | Number of fledglings | -0.31 | -2.89 | 37 | 0.006 |
|  | Tarsus length | 0.81 | 9.75 | 25 | <0.001 |
|  | Year: 2009 | -0.05 | -0.13 | 37 | 0.893 |
|  | Year: 2010 | 0.09 | 0.28 | 37 | 0.783 |
