# Supplementary material for: Early Life Events Carry Over to Influence Pre-Migratory Condition in a Free-Living Songbird
Source: PLoS One. 2011 Dec 16;6(12):e28838. doi: 10.1371/journal.pone.0028838 (PMC3241683; doi:10.1371/journal.pone.0028838)
Supplement: Table S10 — Results from experimental brood manipulations when nestling that received PBS are (1) included and (2) excluded (n = 5). A random effect was included for natal nest. Reference level for year is 2009. Parameter estimates based on un-standardized data. (DOC) [file pone.0028838.s014.doc]

| **Model** | **Model Term** | **β** | **t** | **df** | **P (t)** |
| --- | --- | --- | --- | --- | --- |
| (1) Dataset including nestlings that received PBS in 2009 | Treatment: reduced | 1.20 | 3.68 | 35 | 0.001 |
|  | Timing of nesting | -0.01 | -1.01 | 35 | 0.321 |
|  | Tarsus length | 1.04 | 11.49 | 95 | <0.001 |
|  | Year: 2010 | 0.16 | 0.51 | 35 | 0.616 |
| (2) Dataset excluding nestlings that received PBS in 2009 | Treatment: reduced | 1.16 | 3.57 | 35 | 0.001 |
|  | Timing of nesting | -0.01 | -0.89 | 35 | 0.380 |
|  | Tarsus length | 1.05 | 10.15 | 90 | <0.001 |
|  | Year: 2010 | 0.14 | 0.44 | 35 | 0.664 |
