# Supplementary material for: Early Life Events Carry Over to Influence Pre-Migratory Condition in a Free-Living Songbird
Source: PLoS One. 2011 Dec 16;6(12):e28838. doi: 10.1371/journal.pone.0028838 (PMC3241683; doi:10.1371/journal.pone.0028838)
Supplement: Table S11 — Model results (interactions only; no main effects) for hypothesized two-way interactions. ‘X’ denotes an interaction. The moult progression X year interaction for fat mass was no longer significant after the other interactions were removed from the model (year: 2009: β = −0.54, t = −1.83, DF = 47, P = 0.07; year: 2010: β = −0.02, t = −0.11, DF = 47, P = 0.91). Also, because the effect size and significance of interaction terms for date captured and year were similar in 2009 and 2010 for both total body water and fat mass, year was recoded as ‘2008’ and ‘2009+2010’ in order to reduce the number of parameters and paths included in the final path model. Random effects were included for individual nested within natal nest. Parameter estimates based on standardized data. (DOC) [file pone.0028838.s015.doc]

| **Model** | **Model Term** | **β** | **t** | **df** | **P (t)** |
| --- | --- | --- | --- | --- | --- |
| (1) Number of fledglings | Timing of nesting X year: 2009 | -0.35 | -0.92 | 54 | 0.362 |
|  | Timing of nesting X year: 2010 | 0.21 | 0.80 | 54 | 0.426 |
| (2) Nestling mass | Timing of nesting X year: 2009 | 0.09 | 0.25 | 50 | 0.801 |
|  | Timing of nesting X year: 2010 | 0.03 | 0.12 | 50 | 0.906 |
|  | Number of fledglings X year: 2009 | 0.26 | 1.07 | 50 | 0.291 |
|  | Number of fledglings X year: 2010 | 0.04 | 0.19 | 50 | 0.848 |
|  | Timing of nesting X number of fledglings | 0.09 | 1.18 | 50 | 0.242 |
| (3) Total body water | Nestling mass X timing of nesting | -0.03 | -0.31 | 25 | 0.758 |
|  | Nestling mass X year: 2009 | 0.11 | 0.42 | 25 | 0.676 |
|  | Nestling mass X year: 2010 | -0.08 | -0.38 | 25 | 0.705 |
|  | Nestling mass X date captured | 0.04 | 0.50 | 42 | 0.619 |
|  | Timing of nesting X moult progression | 0.01 | 0.04 | 42 | 0.965 |
|  | Timing of nesting X date captured | 0.17 | 2.07 | 42 | 0.044 |
|  | Timing of nesting X year: 2009 | -0.39 | -0.99 | 42 | 0.324 |
|  | Timing of nesting X year: 2010 | -0.08 | -0.27 | 44 | 0.790 |
|  | Moult progression X date captured | 0.08 | 0.69 | 44 | 0.493 |
|  | Moult progression X year: 2009 | -0.07 | -0.22 | 42 | 0.829 |
|  | Moult progression X year: 2010 | -0.04 | -0.17 | 42 | 0.864 |
|  | Date captured X year: 2009 | -0.88 | -3.14 | 42 | 0.003 |
|  | Date captured X year: 2010 | -0.81 | -3.85 | 42 | <0.001 |
| (4) Fat mass | Nestling mass X timing of nesting | -0.03 | -0.43 | 25 | 0.674 |
|  | Nestling mass X year: 2009 | 0.27 | 1.04 | 25 | 0.308 |
|  | Nestling mass X year: 2010 | 0.13 | 0.64 | 25 | 0.526 |
|  | Nestling mass X date captured | 0.03 | 0.36 | 43 | 0.719 |
|  | Timing of nesting X moult progression | 0.04 | 0.27 | 43 | 0.790 |
|  | Timing of nesting X date captured | 0.17 | 1.69 | 43 | 0.098 |
|  | Timing of nesting X year: 2009 | -0.22 | -0.54 | 44 | 0.591 |
|  | Timing of nesting X year: 2010 | -0.11 | -0.37 | 44 | 0.714 |
|  | Moult progression X date captured | 0.09 | 0.62 | 43 | 0.537 |
|  | Moult progression X year: 2009 | -0.73 | -2.02 | 43 | 0.049 |
|  | Moult progression X year: 2010 | -0.15 | -0.53 | 43 | 0.601 |
|  | Date captured X year: 2009 | 0.79 | 2.27 | 43 | 0.028 |
|  | Date captured X year: 2010 | 0.53 | 2.05 | 43 | 0.047 |
| (5) Moult progression | Timing of nesting X year: 2009 | 0.23 | 0.66 | 44 | 0.514 |
|  | Timing of nesting X year: 2010 | 0.24 | 1.28 | 44 | 0.208 |
|  | Date captured X year: 2009 | 0.07 | 0.41 | 50 | 0.683 |
|  | Date captured X year: 2010 | -0.12 | -0.87 | 50 | 0.388 |
|  | Timing of nesting X date captured | -0.07 | -1.19 | 50 | 0.237 |
