# Supplementary material for: Early Life Events Carry Over to Influence Pre-Migratory Condition in a Free-Living Songbird
Source: PLoS One. 2011 Dec 16;6(12):e28838. doi: 10.1371/journal.pone.0028838 (PMC3241683; doi:10.1371/journal.pone.0028838)
Supplement: Table S12 — Factors affecting: (1) Nestling mass, (2) pre-migratory total body water, (3) pre-migratory fat mass, and (4) pre-migratory moult progression. For number 1, a random effect was included for natal nest. For numbers 2 through 4, random effects were included for individual nested within natal nest. Reference level for year is 2008. 2 represents curvilinear term and ‘X’ denotes an interaction. Parameter estimates based on un-standardized data. (DOC) [file pone.0028838.s016.doc]

| **Model** | **Model Term** | **β** | **t** | **df** | **P (t)** |
| --- | --- | --- | --- | --- | --- |
| (1) Nestling mass | Timing of nesting | -0.04 | -3.83 | 55 | <0.001 |
|  | Number of fledglings | -0.65 | -3.56 | 55 | <0.001 |
|  | Tarsus length in nest | 1.04 | 8.93 | 51 | <0.001 |
|  | Year: 2009 | 0.53 | 1.38 | 55 | 0.174 |
|  | Year: 2010 | 0.66 | 2.21 | 55 | 0.032 |
| (2) Pre-migratory total body water (g) | Nestling mass | 0.18 | 1.93 | 28 | 0.064 |
|  | Timing of nesting | 0.01 | 0.68 | 46 | 0.503 |
|  | Body moult progression | 1.50 | 2.01 | 48 | 0.050 |
|  | Body moult progression^2^ | -0.30 | -2.32 | 48 | 0.024 |
|  | Juvenile tarsus length | 0.23 | 1.37 | 28 | 0.183 |
|  | Time of day captured | 0.04 | 1.16 | 48 | 0.252 |
|  | Date captured | 0.01 | 1.96 | 48 | 0.056 |
|  | Year: 2009 | 1.95 | 4.82 | 46 | <0.001 |
|  | Year: 2010 | -0.33 | -0.99 | 46 | 0.329 |
|  | Timing of nesting X date captured | 0.20 | 2.11 | 48 | 0.040 |
|  | Date captured X year | -0.54 | -5.83 | 48 | <0.001 |
| (3) Pre-migratory fat mass index | Nestling mass | 0.14 | 2.86 | 28 | 0.008 |
|  | Timing of nesting | < -0.01 | -0.85 | 46 | 0.399 |
|  | Body moult progression | 0.10 | 1.24 | 50 | 0.219 |
|  | Juvenile tarsus length | -0.11 | -1.28 | 50 | 0.210 |
|  | Time of day captured | 0.06 | 2.56 | 50 | 0.014 |
|  | Date captured | 0.02 | 3.78 | 50 | <0.001 |
|  | Year: 2009 | -0.56 | -2.88 | 46 | 0.006 |
|  | Year: 2010 | -0.32 | -1.81 | 46 | 0.077 |
|  | Date captured X year | 0.27 | 4.29 | 50 | <0.001 |
| (2) Pre-migratory moult progression | Timing of nesting | -0.02 | -4.02 | 46 | <0.001 |
|  | Date captured | 0.03 | 6.97 | 53 | <0.001 |
|  | Year: 2009 | 0.28 | 1.04 | 46 | 0.302 |
|  | Year: 2010 | 1.10 | 5.08 | 46 | <0.001 |
